# JBO 2024 List of Reviewers

**DOI:** 10.1117/1.JBO.30.1.010102

**Published:** 2025-01-11

## Abstract

Thanks to reviewers who served JBO in 2024.

The *Journal of Biomedical Optics* sincerely thanks the following individuals who served as reviewers in 2024. The success of our publication hinges on the voluntary contributions of time and energy put forth by these professionals.

Elham Abouei
Robert Adamson
Salavat Aglyamov
Vasundhara Agrawal
Alba Alfonso-Garcia
Mark Anastasio
Bhavna Antony
Bahman Anvari
Matthew Applegate
Shahbaz Askari
Kamran Avanaki
Wesley Baker
Santosh Balakrishnan
Timothy Baran
Luca Baratelli
Bernhard Baumann
Paul Beard
Mikhail Berezin
Andrew Berger
David Birch
Kostadinka Bizheva
Richard Blackmon
Selina Bopp
Denis Bragin
David Brenes
Jorge Brieva
J. Quincy Brown
Gijs Buist
David Busch
Alexander Bykov
Refik Mert Cam
Christopher Campbell
Liangcai Cao
Rui Cao
Xu Cao
Stefan Carp
Jeffrey Cassidy
Paloma Casteleiro Costa
Jonathan Celli
Bo-Jui Chang
Shuang Chang
Nanguang Chen
Sung-Liang Chen
Xi Chen
Xiaoxi Chen
Xueli Chen
Yanda Chen
Yu Chen
Philip Cheney
Qian Cheng
Ata Chizari
Regine Choe
Wonshik Choi
Nicol s Coca López
Mark Coughlan
Anna Cleta Croce
Christian Crouzet
Michal Dabrowski
Michael Daly
Behdad Dashtbozorg
Scott C. Davis
Valentin Demidov
Jurre den Haan
Dinesh Dhankhar
Charles DiMarzio
Ana Doblas
Alexander Doronin
Viktor Dremin
Andrey Dunaev
Nicholas Durr
Adam Eggebrecht
Shay Elmalem
Daniel Elson
Olga Eremina
Thomas Ertl
Rasa Eskandari
Muhannad Fadhel
Felix Fanjul-Velez
Joyce Farrell
Jinchao Feng
Ziang Feng
Reto Fiolka
Morgan Fogarty
Anderson Freitas
Daniel Fried
Ekaterina Galanzha
Feng Gao
Gan Gao
Hector Garcia
Irene Georgakoudi
Nirmalya Ghosh
Michael Giacomelli
Aaron Goldfain
Dimitris Gorpas
Debabrata Goswami
Hanxue Gu
Richard Haindl
Zeinab Hajjarian
Andreas Hauptmann
Carole Hayakawa
Da He
Honghui He
Qinghua He
Ahmed Heikal
Marjaneh Hejazi
Bradley Henderson
Maria del Socorro Hernandez-Montes
Trey Highland
Dierck Hillmann
Mohammad Hodaei
Guosong Hong
Xiaoming Hu
Kevin Huang
Luzhe Huang
Yong Huang
Alexsandra Ilina
Inna Ilina
Mengyu Jia
Hanna Jonasson
Myeong Jin Ju
Francis Kalloor Joseph
Farah Kamar
Dongkyun Kang
Karol Karnowski
Kavon Karrobi
Elif Kayaalp Nalbant
Necla Kenar
Gordon Kennedy
Beop-Min Kim
Mikhail Kirillin
Yury Kistenev
Matthias Klemm
Tiffany Ko
Katarzyna Komolibus
Sanathana Konugolu Venkata Sekar
Ivan Kosik
Murali Krishnamoorthy
Nikola Krstajic
Suvvi Kuppur Narayana Swamy
Xiaomin Lai
Jesse Lam
Frederic Lange
Maryse Lapierre-Landry
Kirill Larin
Ethan LaRochelle
Audrey Laurence
V. N. Du Le
Hsiang-Chieh Lee
Kijoon Lee
Rainer Leitgeb
Benjamin Levy
Chunqiang Li
Hui Li
Lei Li
Shuying Li
Ting Li
Zhe Li
Haonan Lin
Weichiang Lin
Norman Lippok
Chengbo Liu
Gangjun Liu
Yang Liu
Yang Liu
Dirk Lorenser
Jie Lu
Felix Lucka
Xiaoyi Lv
Ling Ma
Yayao Ma
Oscar MacCormac
Steen Madsen
Shuichi Makita
Srivalleesha Mallidi
Efthymios Maneas
Teo Manojlovic
Igor Meglinski
Marcello Melis
Rodrigo Menezes Forti
Guanghan Meng
Zhaokai Meng
J. Sven Mieog
David Miller
Eric Miller
Thomas Milner
Jianhua Mo
Guillermo Monroy
Sakina Mohammed Mota
Jenna Mueller
Mircea Mujat
Sabyasachi Mukhopadhyay
Peter Naglic
Amir Nahas
Takeshi Namita
Sreyankar Nandy
John Quan Nguyen
Linyu Ni
Line Nielsen
Izumi Nishidate
Christine O’Brien
Marien Ochoa Mendoza
Joshua Pace
Rahul Pal
Gregory Palmer
Vikas Pandey
YongKeun Park
Simon Parschat
Ashwin Parthasarathy
Nishigandha Patil
Avijit Paul
Vijitha Periyasamy
Alexander Peterson
Arthur Petusseau
T. Joshua Pfefer
Yamuna Phal
Quoc-Hung Phan
Thinh Phan
Daqing Piao
Mark Pierce
Michael Pircher
Raju Poddar
Lisa Poulikakos
Junle Qu
Timothy Quang
Valentina Quaresima
Matin Raayai Ardakani
Rahul Ragunathan
Narasimhan Rajaram
Praveenbalaji Rajendran
Matthew Reed
Marco Renna
Jungtae Rha
Constance Robbins
Andres Rodriguez
Joel Rodriguez
Bernhard Roth
Cody Rounds
Angelika Rueck
Theo Ruers
Rolf Saager
Teemu Sahlstrom
Partha Sahu
Guennadi Saiko
Inga Saknite
Angelo Sassaroli
Travis Sawyer
Allison Scarbrough
Herbert Schneckenburger
Felix Scholkmann
Hinnerk Schulz-Hildebrandt
Augustino Scorzo
Eric Seibel
Kerry Setchfield
Babak Shadgan
Natan Shaked
Anna Sharikova
Arunima Sharma
Ryan Shelton
Douglas Shepherd
Jindou Shi
Lingyan Shi
Mengjie Shi
Garth Simpson
Lagnojita Sinha
Melissa Skala
David Sliney
Thomas Smart
Jason Smith
Zachary Smith
Sanjeev Soni
Janis Spigulis
Keith St. Lawrence
Jost Stergar
Samuel Streeter
Tomas Stromberg
Aleh Sudakou
Chia-Wei Sun
Mingzhu Sun
Ulas Sunar
Yongjin Sung
Qinggong Tang
Yue Tang
M Ali Tavallaei
Michaela Taylor-Williams
Damber Thapa
Chao Tian
Tadej Tomanič
Veronica Torres
Maciej Trusiak
Ying Yin Tsui
Valery Tuchin
Giovanni Ughi
Hafeez Ullah Janjua
Paul Kumar Upputuri
Shikhar Uttam
Sarah Vavrek
Alberto Vazquez
Rudolf Verdaasdonk
Umberto Villa
Fabian Voigt
Sunil Vyas
Hui Wang
Junzhe Wang
Lidai Wang
Lingyun Wang
Wenbo Wang
Yaning Wang
Robert Weersink
Maciej Wrobel
Jingyi Wu
Yicong Wu
Lennard Wurm
Jun Xia
Wenfeng Xia
Xiangheng Xiao
Yijing Xie
Chen Xu
Kexin Xu
Yuan Xu
jiahui xun
Feng Yan
Bin Yang
Euitaek Yang
Fan Yang
Jiamiao Yang
Xinmai Yang
Cuiping Yao
Gang Yao
Jingting Yao
Jian Ye
Filiz Yesilkoy
Jonghee Yoon
Linhui Yu
Weiming Yu
Jie Yuan
Shuhua Yue
Mark Zarella
Haishan Zeng
Jiong Zhang
Limin Zhang
Shuangyang Zhang
Yi Zhang
Jianhua Zhao
Jie Zhao
Mengyang Zhao
Yanyu Zhao
Guoan Zheng
Chao Zhou
Caigang Zhu
Jiyun Zhu
Shouping Zhu
Yihua Zhu
Yun Zou
Chao Zuo

